# Combinatorial effects of zinc deficiency and arsenic exposure on zebrafish (*Danio rerio*) development

**DOI:** 10.1371/journal.pone.0183831

**Published:** 2017-08-24

**Authors:** Laura M. Beaver, Lisa Truong, Carrie L. Barton, Tyler T. Chase, Greg D. Gonnerman, Carmen P. Wong, Robert L. Tanguay, Emily Ho

**Affiliations:** 1 Biological and Population Health Sciences, Oregon State University, Corvallis, Oregon, United States of America; 2 Linus Pauling Institute, Oregon State University, Corvallis, Oregon, United States of America; 3 Department of Environmental and Molecular Toxicology, Sinnhuber Aquatic Research Laboratory, Oregon State University, Corvallis, Oregon, United States of America; 4 The Environmental Health Sciences Center, Oregon State University, Corvallis, Oregon, United States of America; 5 Center for Genome Research and Biocomputing, Oregon State University, Corvallis, Oregon, United States of America; 6 Moore Family Center for Whole Grain Foods, Nutrition and Preventive Health, Oregon State University, Corvallis, Oregon, United States of America; Hong Kong University of Science and Technology, CHINA

## Abstract

Zinc deficiency and chronic low level exposures to inorganic arsenic in drinking water are both significant public health concerns that affect millions of people including pregnant women. These two conditions can co-exist in the human population but little is known about their interaction, and in particular, whether zinc deficiency sensitizes individuals to arsenic exposure and toxicity, especially during critical windows of development. To address this, we utilized the *Danio rerio* (zebrafish) model to test the hypothesis that parental zinc deficiency sensitizes the developing embryo to low-concentration arsenic toxicity, leading to altered developmental outcomes. Adult zebrafish were fed defined zinc deficient and zinc adequate diets and were spawned resulting in zinc adequate and zinc deficient embryos. The embryos were treated with environmentally relevant concentrations of 0, 50, and 500 ppb arsenic. Arsenic exposure significantly reduced the amount of zinc in the developing embryo by ~7%. The combination of zinc deficiency and low-level arsenic exposures did not sensitize the developing embryo to increased developmental malformations or mortality. The combination did cause a 40% decline in physical activity of the embryos, and this decline was significantly greater than what was observed with zinc deficiency or arsenic exposure alone. Significant changes in RNA expression of genes that regulate zinc homeostasis, response to oxidative stress and insulin production (including *zip1*, *znt7*, *nrf2*, *ogg1*, *pax4*, and *insa*) were found in zinc deficient, or zinc deficiency and arsenic exposed embryos. Overall, the data suggests that the combination of zinc deficiency and arsenic exposure has harmful effects on the developing embryo and may increase the risk for developing chronic diseases like diabetes.

## Introduction

Zinc deficiency among pregnant women is a public health concern because zinc is an essential micronutrient that supports normal growth and development during pregnancy and childhood [1–9]. As many as 82% of pregnant women worldwide have inadequate zinc uptake [10]. Maternal zinc deficiency may have adverse consequences for their offspring both acutely during pregnancy and through their lifespan, by increasing their susceptibility to diseases as an adult. For example, suboptimal zinc consumption in humans was associated with increased premature birth, low birthweights, and increased congenital malformations which are all possible acute effects of zinc deficiency [2–9]. Later in life, marginal maternal zinc deficiency in rats has been associated with decreased weight, increased percentage of body fat, and impaired insulin secretion in the offspring [11]. Zinc is required for many biological processes in the body including insulin synthesis and storage (reviewed in [12]). Young patients with type 2 diabetes mellitus have significantly lower zinc levels, suggesting that the zinc status of the individual may play a role in the susceptibility of developing type 2 diabetes [13]. Zinc also plays a protective role in the body, acting as a pro-antioxidant and cofactor for many enzymes that regulate the response to reactive oxygen species [14]. Zinc deficiency causes increased oxidative stress and is associated with increased damage of DNA, proteins and lipids [15]. Together these studies illustrate that embryonic zinc deficiency could make individuals more susceptible to long term chronic diseases.

The impact of environmental pollutant exposures during pregnancy is also a major public health concern because they can increase the risk of adverse health effects for the fetus [16]. Inorganic arsenic (hence forth referred to as arsenic) is of particular concern because pregnant mothers in at least 20 different countries (including Bangladesh, Taiwan, Mexico, Chile, Argentina, Vietnam, Laos, India, China, Romania, and the United States) live above aquifers with arsenic levels that exceed the World Health Organizations drinking water recommendation which is 10 μg / liter [17, 18]. Long-term exposure to arsenic in adults occurs primarily through consumption of contaminated drinking water and food, and is associated with skin disorders, increased cancer incidence, high blood pressure, and increased risk for diabetes [19, 20]. At the molecular level, arsenic exposure causes oxidative stress, and this is thought to contribute to disease formation [21]. Acute adverse effects of arsenic exposure during pregnancy have been found in many population-based studies [22–26]. More specifically, increased arsenic exposure was associated with increased risk of spontaneous abortion, still birth, infant mortality, and a significant reduction in birth weight. Exposure to arsenic during pregnancy and childhood is also associated with increased occurrence and/or severity of lung disease, cardiovascular disease, and cancer, and there are decades-long latency periods reported between exposure and disease appearance (reviewed in [27, 28].

Zinc deficiency and arsenic exposures often co-exist in the human population because millions of people are exposed unsafe levels of arsenic in their drinking water, and a high number of the pregnant mothers in these regions are unlikely to consume the recommended amounts of zinc [10, 18]. While we know that zinc deficiency and arsenic exposure have some common pathological targets, such as increasing the risk of mortality to the developing fetus, the effect of zinc status on arsenic related toxicity is unknown, particularly during critical windows of development. To address this gap in knowledge, we tested the hypothesis that parental zinc deficiency sensitizes the developing embryo to low-level arsenic exposures, leading to altered developmental outcomes. We tested the hypothesis in *Danio rerio* (Zebrafish), which is a premier model for both development and environmental toxicant exposure [29]. More specifically, zebrafish are a small, complex vertebrate organism with rapid development and a short life cycle [30]. The embryos develop externally and are optically clear to allow for non-invasive assessments. It is estimated that 99% of embryonic-essential fish genes are homologues in human embryonic development [31, 32]. Importantly, like humans, zebrafish require dietary zinc intake and have similar regulatory mechanisms that maintain zinc homeostasis including its uptake and intracellular distribution by zinc importers (*zip*/*slc39*) and zinc exporters (*znt*/*slc30*) [33]. We recently developed a zinc deficient zebrafish diet and demonstrated the embryos from zinc deficient zebrafish parents are also zinc deficient, and this is associated with changes in gene expression, decreased physical activity, and a 2-fold increase in mortality in the embryos [34].

Herein we utilized the zebrafish model and evaluated the effects of zinc deficiency and environmentally relevant concentrations of arsenic exposure on development. More specifically, we determined if these variables, alone and in combination, altered the activity of the embryo, and increased the incidence of morbidity and mortality. We also evaluated the extent to which zinc deficiency and/or arsenic exposure altered the expression of key genes that regulate stress response and insulin production in the developing embryo. Additionally, embryos exposed to environmentally relevant concentrations of arsenic were evaluated for changes in zinc homeostasis.

## Material and methods

### Fish husbandry and diet preparation

This study was carried out in strict accordance with the recommendations in the Guide for the Care and Use of Laboratory Animals of the National Institutes of Health in accordance with protocols approved by the Oregon State University Institutional Animal Care and Use Committee (IACUC). Wild type Tropical 5D zebrafish (5D) were raised and maintained at the Sinnhuber Aquatic Research Laboratory (SARL) at Oregon State University. All adult animals were fed standard lab diet (Gemma Micro. Skretting, Tooele, France) until 8 weeks post fertilization when they were moved to clean enclosures, randomized to either zinc deficient or zinc adequate diets, and housed at densities of ~6 fish / liter as previously described [34]. The zebrafish diets were produced as previously described [34]. Feeding volumes for all feeds were ~5% body weight / day, or until satiation, given over 2–3 feedings in a day depending on life stage. To produce zinc adequate and zinc deficient embryos, parental adult fish were fed zinc adequate (control) or zinc deficient diet for at least 8 weeks. These adult fish were then spawned to produce zinc adequate and zinc deficient embryos. Spawning occurred in small groups at an interval of every 2–3 weeks. To perform embryonic arsenic exposures, embryo chorions from each dietary group were enzymatically removed using pronase (83 μL of 25.3 U/mg; Roche, Indianapolis, IN, USA) at 4 hours post fertilization (hpf) using a custom automated dechorionator and protocol described in Mandrell et al. [35]. Dechorionated embryos were placed in individual wells of a 96-well plate (Falcon U-bottom; supplier number: 353227) with 90 μL of E2 embryo media. At 6 hpf, embryos were exposed with 10 μL of embryo media, or varying concentrations sodium arsenite (NaAsO_2_, Sigma-Aldrich) resulting in final well concentrations of 0, 50 or 500 ppb of arsenic. These concentrations were chosen because 50 ppb arsenic was the limit set by the Environmental Protection Agency for drinking water up until 2001. Furthermore, arsenic can still be found at the 50–500 ppb concentrations in various ground water sources around the world [36]. Embryos were statically exposed until 120 hpf and utilized the nutrition available in the yolk sac and did not need to be fed any diet. For sample collections adult fish or embryos were euthanized with an overdose of the anesthesia drug tricaine mesylate, and all efforts were made to minimize suffering.

### Metal analysis

Zinc, copper, iron, selenium, calcium and magnesium, essential metals in human health were evaluated in diets, adult parental fish, and embryos using Prodigy High Dispersion Inductively Coupled Plasma-Optical Emissions Spectroscopy (ICP-OES) (Teledyne Leeman Labs, Hudson, NH, USA) as previously described [37]. Samples of homogenized adult fish (100 μl from a 2 ml homogenized volume in phosphate-buffered saline), 30 fish embryos, or ~50 mg of diet were digested overnight in 70% ultrapure nitric acid and then diluted 10-fold with Chelex-treated nanopure water. Additionally, fish water was tested periodically using the same ICP-OES technique and shown to have no detectable zinc where the detection limit was 0.5 ng / mL. Samples were analyzed against known metal standards (Ultra Scientific, Kingstown, RI).

### Gene expression

Expression of genes related to zinc transport, metal homeostasis, stress response, and insulin production were evaluated by quantitative real-time PCR. In each experiment total RNA was collected at 0, 6, 24, 48 and 120 hpf from 15–20 pooled embryos / treatment group, and 3–4 biological replicates for each treatment. Embryos were homogenized in 500 μL of RNAzol RT (Sigma-Aldrich, St. Louis, MO), with 0.5 mm zirconium oxide beads and a bullet blender (Next Advance, Averill Park, NY). RNA samples were then purified with Direct-zol RNA MiniPrep kit (Zymo Research, Irvine, CA) following manufactures recommendations. cDNA was synthesized using 250 ng of total RNA and SuperScript III First-Strand Synthesis SuperMix (Thermo Fisher Scientific, Eugene OR). Real time PCR was done using zebrafish-specific primers (S1 Table) and Fast SYBR Green Mastermix (Thermo Fisher Scientific) on a 7900HT Fast Real-Time PCR System (Applied Biosystems, Foster City, CA) as previously described [34]. PCR conditions were programmed as follows: 95°C for 20 s, followed by 40 cycles of denaturing at 95°C for 1 s, annealing and extension at 58°C for 20 s, followed by a dissociation curve at 95°C for 15 s, 60°C for 15 s, and 95°C for 15 s. A dilution series of 10^3^, 10^4^, 10^5^, 10^6^, and 10^7^ copies of template DNA served as internal standard for quantification [38]. In order to normalize and control for changes in gene expression during development, the copy number of the gene of interest was divided by the copy number of the housekeeping gene (*odc1*), and then expressed relative to the mean level found at the same time point in zinc adequate embryos exposed to no arsenic.

### Developmental toxicity evaluation

At 24 and 120 hpf, embryos were assessed for developmental toxicity endpoints as described in Truong et al. [39]. Briefly, 4 endpoints were observed at 24 hpf, and 18 separate morphological evaluations were conducted at 120 hpf. Each endpoint was scored in a binary fashion (present/absent) and collected using a custom zebrafish laboratory integration system (Zebrafish Acquisition and Analysis Program; ZAAP). Statistical analysis was performed using R code from the methodology described in Truong et al., which is a binomial test that calculates lowest effect levels (LELs) for each endpoint to identify incidences that exceed a significance threshold above that endpoints control [40, 41].

### Larval photomotor response assay

To determine if zinc deficiency and arsenic exposure alters the activity of embryos following light and dark stimuli the photomotor response assay was performed. Embryos at 120 hpf were tested in the 96-well exposure plates by placing them into the Viewpoint Zebrabox HD (software version 3.2, Viewpoint Life Sciences, Lyon, France) and measuring locomotor activity using the tracking setting during 3 minute periods of alternating light and dark for a total of 24 minutes. The integration time was set to 6 seconds to increase statistical power. The total movement (swim distance) in response to the multiple light-dark transition was tracked by an HD camera at 30 frames / s [41]. Any dead or malformed animals at 120 hpf were excluded from the data analysis and 232–329 embryos were analyzed. Raw data files were processed using custom R scripts to average the total distance traveled for each integration time point, and then the area under the curve was computed [41]. The overall area under the curve was compared to the control (zinc adequate, no arsenic) using a Kolmogorov-Smirnov test (p<0.01).

## Results

### Effect of arsenic exposure and zinc deficiency on zinc levels and expression of genes that regulate metal homeostasis

**Fig 1:**
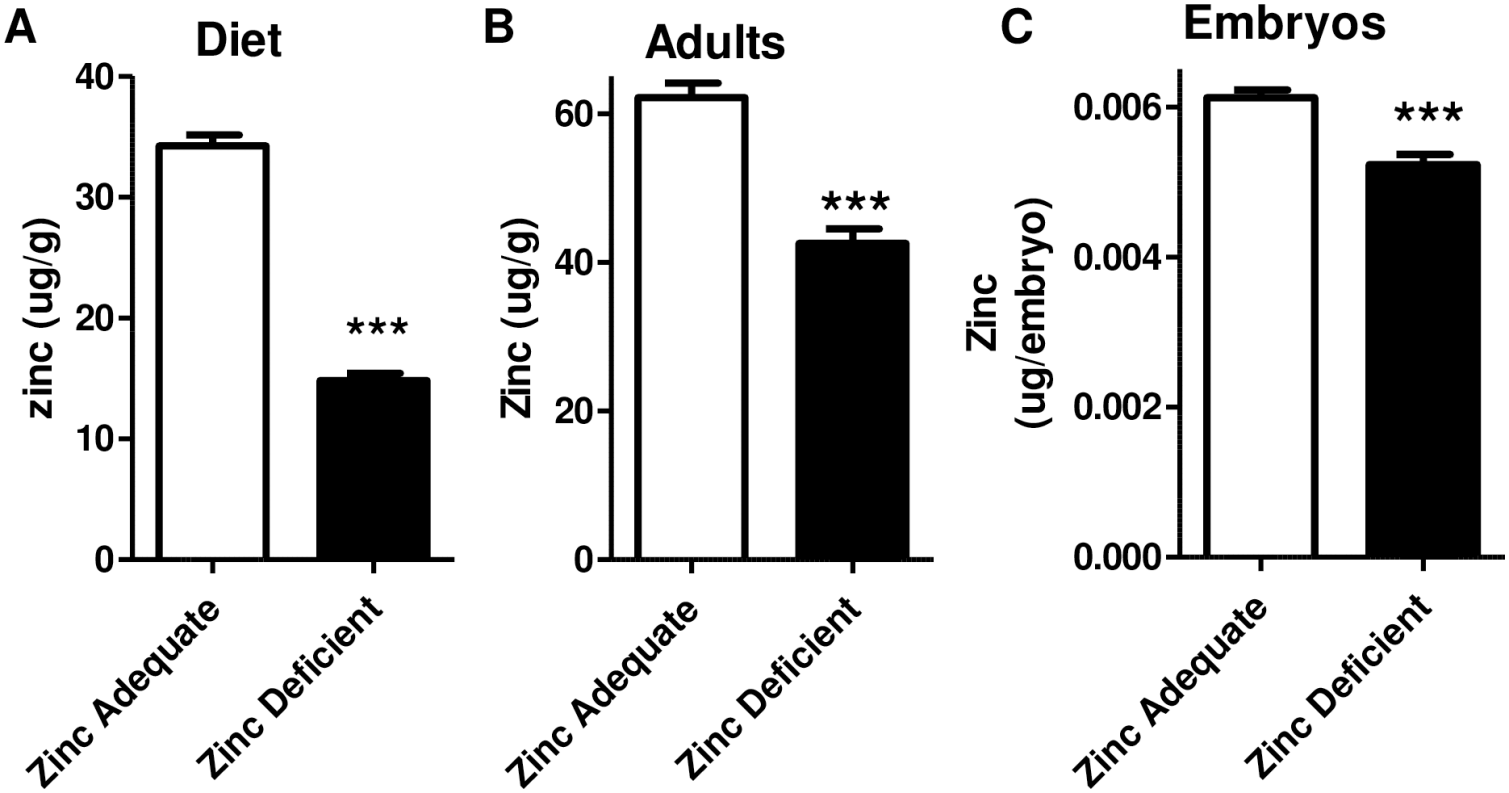
Adult zebrafish fed a zinc deficient diet have zinc deficient offspring. Bars indicate the average amount (± SEM) of zinc, as measured by ICP-OES, in A) fish diet B) adult fish and C) embryos produced by the adult fish. B) Data was obtained on fish that were 4–6 months old and had been on the diet for at least 2 months. C) Embryos were 120 hpf at time of analysis. A-C) Data is representative of at least three independent experiments and n = 24 for diet, n = 35 for adults, and n = 34 for embryos. Significant differences between the zinc adequate and zinc deficient samples were calculated using t-tests and *** indicate significant differences between the groups where p < 0.001 respectively.

Fish were fed either a zinc adequate diet that contained 33.81 μg of zinc per gram of diet, or a zinc deficient diet that was identical in composition and mineral content from the control except that it had 14.45 μg of zinc per gram of diet (Fig 1A and S2 Table). Adult fish fed the zinc deficient diet for more than 4 weeks had a significant 31.5% reduction in the amount of zinc found per gram of body weight (Fig 1B) and zinc depletion with the zinc deficient diet was equal in both genders (data not shown). There was no difference between the zinc adequate and zinc deficient adults in the amount of other essential metals including calcium, iron, magnesium and selenium (S2 Table). Progeny born of zinc deficient parents had 15% less zinc than what was found in control embryos (Fig 1C), and the amount of calcium, copper, iron, magnesium, or selenium detected between control and zinc deficient embryos were not different (S2 Table).

**Fig 2:**
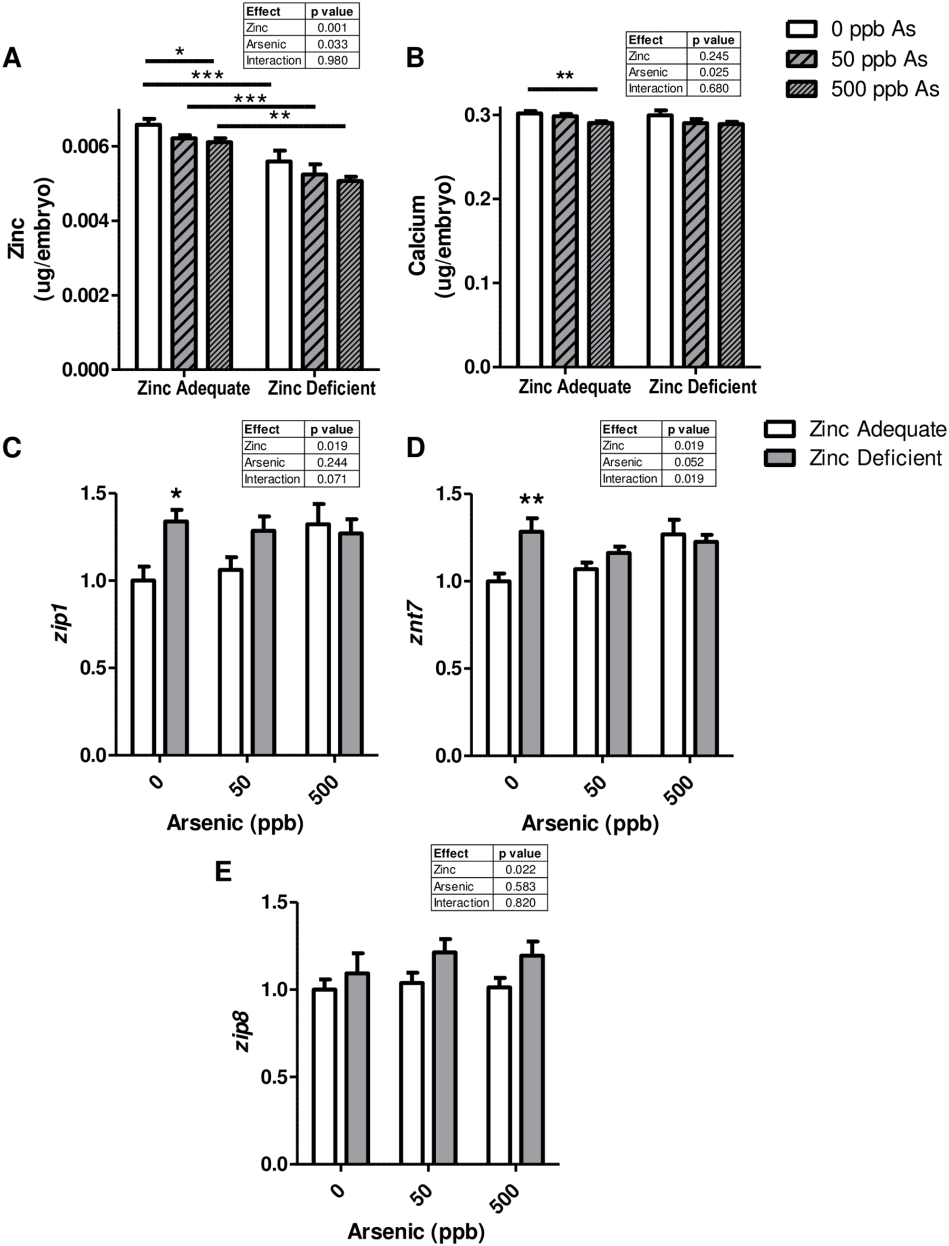
Arsenic exposure decreases zinc and calcium in embryos and zinc deficiency and arsenic effects *zip1*, *znt7* and *zip8* mRNA levels. A-B) Bars are indicative of the mean (± SEM) amount of A) zinc, or B) calcium, as determined by ICP-OES, in zinc adequate or zinc deficient embryos continuously exposed to 0, 50, or 500 ppb arsenic starting at 6 hpf and collected at 120 hpf. Data is representative of at least three independent experiments and n = 9–12. C-E) Bars represent mean (± SEM) mRNA levels of indicated zinc transporters at 120 hpf in zinc adequate (white bars) or zinc deficient (grey bars) embryos exposed to 0, 50, or 500 ppb arsenic. Data represent an average of 7–8 replicates per treatment group and were obtained from 2 independent experiments. A-E) Significant differences between samples were calculated using two-way ANOVAs or one way ANOVAs as appropriate with Bonferroni post-tests. For post-tests *, **, and *** indicate significant differences between the groups where p < 0.05, p < 0.01, and p < 0.001 respectively.

We exposed control and zinc deficient embryos to environmentally relevant concentrations of arsenic (0, 50, or 500 ppb) and evaluated the amount of various trace elements at 120 hpf. We confirmed the zinc deficient embryos had less zinc than the zinc adequate controls (Fig 2A). We also found that zinc adequate embryos treated with arsenic had a significant decline in the amount of zinc found in the embryos (Fig 2A). To understand the extent of the decline with arsenic treatment we compared the amount of zinc in the embryos exposed to 0 and 500 ppb arsenic and found a 7% and 9.4% reduction in the zinc adequate and zinc deficient embryos respectively. There was also a small, but significant decrease in the amount of calcium in arsenic exposed zinc adequate embryos, which was decreased by 3.8% when comparing at 0 and 500 ppb arsenic treatment in zinc adequate embryos (Fig 2B). Arsenic exposure did not significantly alter the amount of copper, iron, magnesium, and selenium in the embryos (S1 Fig).

To better understand the possible mechanisms by which zinc levels were decreasing in embryos with arsenic exposure, and identify any mechanistic interactions between zinc deficiency and arsenic, we evaluated the effect of both zinc deficiency and arsenic exposure on the expression of genes that regulate metal homeostasis. More specifically, 18 zinc transporters and the metal-regulatory transcription factor 1 (MTF-1) were examined at the mRNA level. Zinc deficient embryos had a significant increase in *zip1* and *znt7* mRNA levels compared to zinc adequate controls (Fig 2C and 2D). This significant difference was not observed in embryos with arsenic exposure because *zip1* and *znt7* expression was increased in zinc adequate fish with arsenic exposure, but did not significantly change with arsenic exposure in zinc deficient embryos. Additionally, two-way ANOVA analysis indicated that *zip8* mRNA levels were elevated in zinc deficient embryos, but there was no significant difference between the zinc adequate and zinc deficient embryos with arsenic exposure (Fig 2E). *zip3*, *zip4*, *zip6*, *zip7*, *zip9*, *zip10*, *zip11*, *zip13*, *znt1*, *znt2*, *znt4*, *znt5*, *znt6*, *znt8*, *znt9* and *mtf-1* mRNA levels were not altered with zinc deficiency, arsenic exposure, nor any combination tested (S2 and S3 Figs).

### Effect of arsenic exposure and zinc deficiency on development

**Fig 3:**
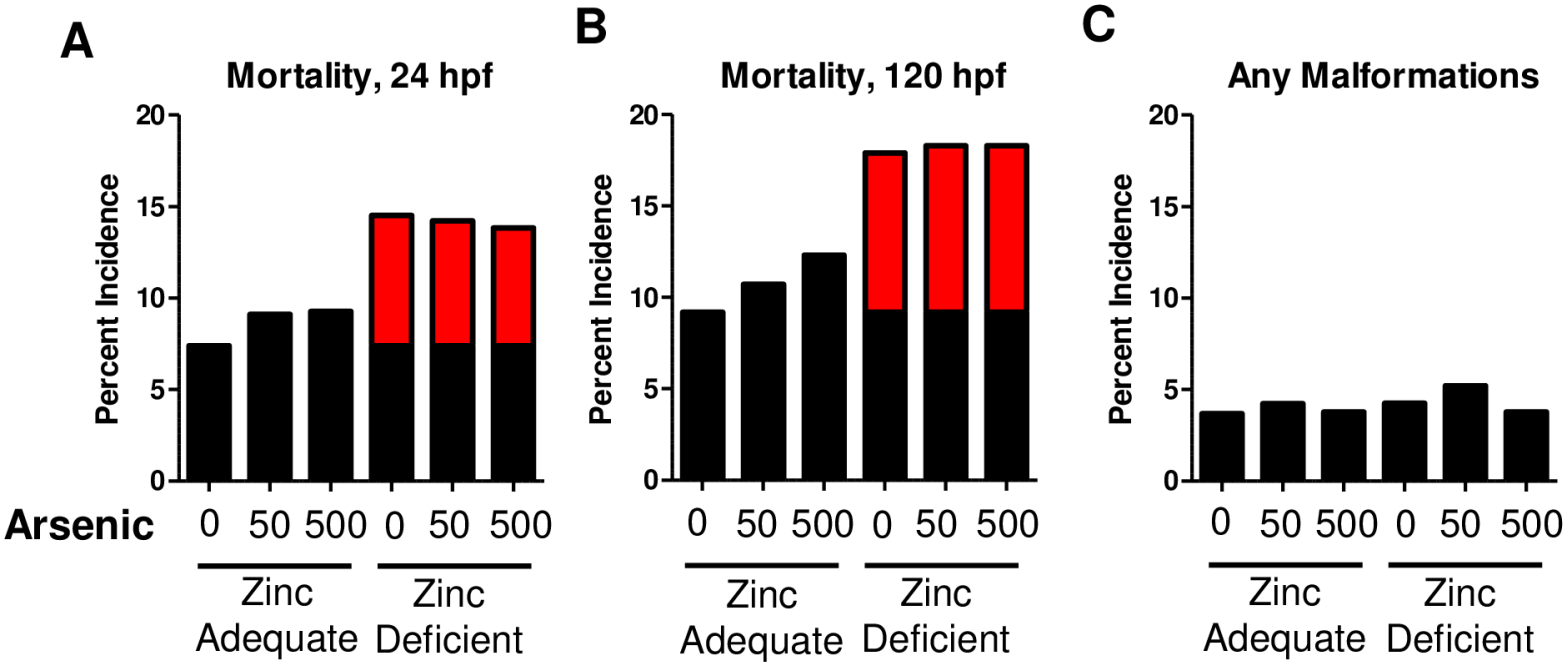
Combination of zinc deficiency and arsenic exposure did not increase mortality or developmental malformations in embryos. A-B) Bars represent average mean developmental malformations and mortality for 445–476 embryos analyzed over 4 independent experiments. Significant differences between groups were calculated with a binomial test that calculates lowest effect levels (LELs) for each endpoint. Red bars indicate significant increases in A-B) mortality, and C) any malformations compared to zinc adequate embryos exposed to 0 ppb arsenic. The same statistical approach was used to evaluate any significant effect of arsenic specifically in zinc deficient embryos and no significant difference was found.

Exposure of embryos to both 50 and 500 ppb arsenic did not significantly increase the incidence of mortality or any developmental malformation in zinc adequate fish (Fig 3A–3C). Arsenic exposure at these concentrations also did not significantly induce any specific developmental malformation examined (S4 Fig). As previously reported, zinc deficient embryos did have a significant increase in mortality compared to zinc adequate controls (Fig 3A and 3B), but arsenic exposure did not further increase the incidence of mortality or developmental malformations in the zinc deficient embryos (Fig 3A–3C and S4 Fig).

**Fig 4:**
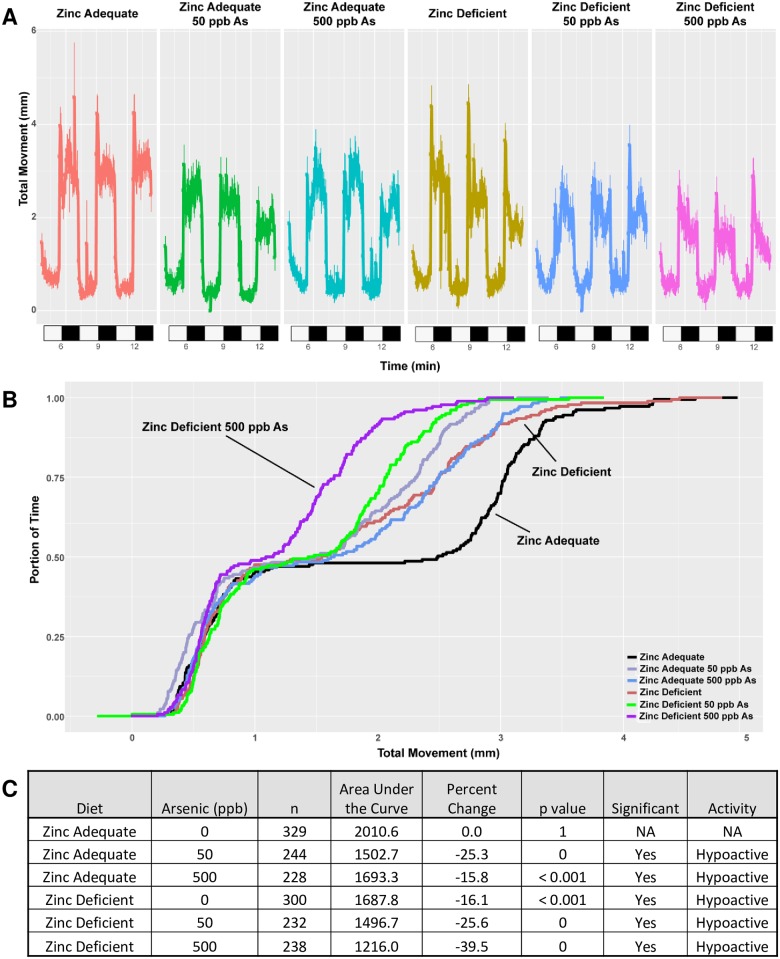
Combination of zinc deficiency and arsenic exposure significantly impact larval behavior. Embryos were collected from zinc adequate or zinc deficient fish, exposed to 0, 50, or 500 ppb arsenic from 6–120 hpf and analyzed for larval activity. Locomotor activity of embryos at 120 hpf were measured by larval photomotor response assay and all data come from at least four independent experiments n = 228–329. A) Indicates movement (y-axis) over time where light conditions during the assay changed and are indicated by white (light) or black (dark) bars on the x-axis. B) A cumulative distribution plot illustrating the total distance moved over time. C) The overall area under the curve for movement was compared using a Kolmogorov-Smirnov test. The condition was considered significant if the P value was less than 0.01 and the percent change was greater than 10%.

We then investigated if zinc status and arsenic exposure altered movement of the embryos using a locomotor activity assay where larval fish (120 hpf) were exposed to repeated cycles of light and dark stimulus. A typical control response is moderate movement while in the light and increased movement in the dark (Fig 4A). Offspring of zinc deficient fish moved during the dark phases but their total movement was 16% less, as shown by the decreased height of the three peaks. Arsenic exposure also significantly decreased the activity in zinc adequate embryos by 15–25%. The hypoactivity of zinc deficient or arsenic treated fish is most apparent when comparing the total movement of the fish over time, where the area under the curve is significantly different between the two groups (Fig 4B and 4C). Interestingly, 50 ppb arsenic exposure in zinc adequate embryos caused a greater decline in overall activity than 500 ppb arsenic suggesting a possible non-monotonic dose response, but these doses were not significantly different from each other (p = 0.061). Combination of zinc deficiency and arsenic exposure decreased the activity of the embryos the most, with a 25.6% decline in activity at the 50 ppb concentration, and a 39.5% decline at the 500 ppb concentration (Fig 4A–4C). This arsenic effect on activity in zinc deficient embryos was significant when compared to either unexposed zinc adequate or unexposed zinc deficient samples (p values < 0.001). The decrease in activity observed with all combinations of zinc deficiency and/or arsenic exposure was caused by a significant decline in activity during the dark phase of the assay when larvae are expected to be active (S3 Table).

### Effect of zinc status and arsenic exposure on expression of genes that regulate stress response and insulin production

**Fig 5:**
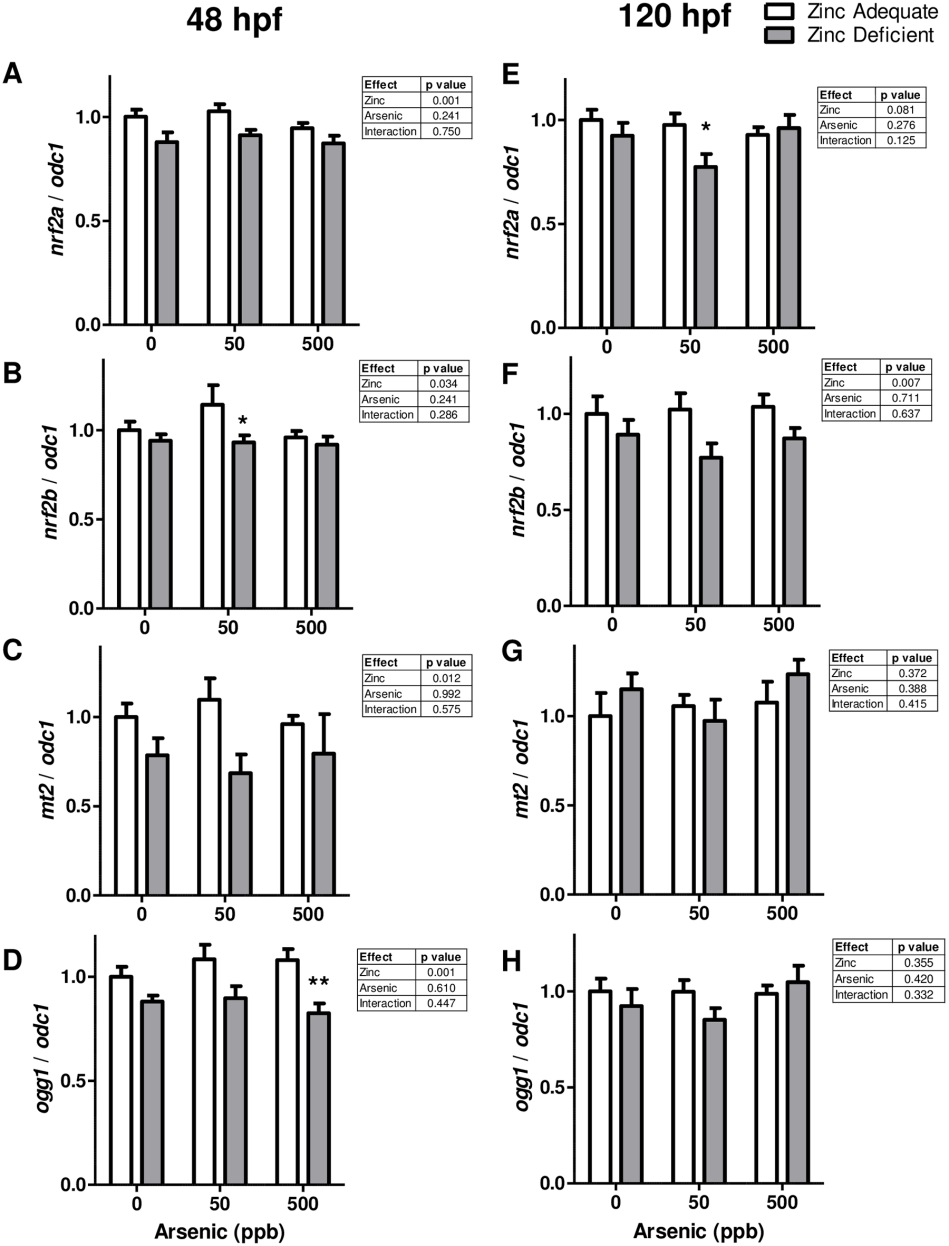
Effect of zinc deficiency and arsenic exposure on the mRNA levels of genes related to oxidative stress. Bars represent mean (± SEM) mRNA levels of the indicated genes in zinc adequate (white bars), or zinc deficient (grey bars) embryos exposed to 0, 50, or 500 ppb arsenic. RNA was collected at 48 (A-D) and 120 (E-H) hpf. Data are from 2–3 independent experiments where (A-D) n = 9, or (E-H) n = 7–8. Significant differences between samples were calculated using two-way ANOVAs with results detailed in individual tables for each gene and time point. Bonferroni post-tests were used to determine differences between zinc adequate and zinc deficient embryos at a given arsenic dose where * and ** indicate significant differences between the groups where p < 0.05 and p < 0.01 respectively.

Arsenic exposure and zinc deficiency independently increase oxidative stress in cell and animal models [21, 42, 43]. For this reason, we evaluated the expression of a few important genes that regulate stress response in the embryos at 48 and 120 hpf. We examined the expression of *nuclear factor (erythroid-derived 2)-like 2* (*nrf2*), *glutamate-cysteine ligase catalytic subunit* (*gclc*), *heme oxygenase 1* (*hmox1*), *metallothionein 2* (*mt2*), *8-oxoguanine DNA glycosylase* (*ogg1*) genes. Nrf2 is a transcription factor that regulates the transcription of genes that act as antioxidant proteins and protect against oxidative damage and zebrafish have duplicate *nrf2* genes (*nrf2a* and *nr2b*) which are co-orthologs of human *NRF2* [44]. *gclc* and *hmox1* are genes that are induced when the Nrf2 pathway is activated. Mt2 is a metal binding protein that both protects cells from exposure to oxidants and regulates zinc levels and distribution. *ogg1* encodes the enzyme responsible for the excision of 8-oxoguanine from the DNA, a mutagenic base produced by exposure to reactive oxygen species. At 48 hpf *nrf2a*, *nrf2b*, *mt2*, and *ogg1* were all significantly suppressed in zinc deficient embryos (Fig 5A–5D). *nrf2b* was significantly different between zinc adequate and zinc deficient embryos exposed to 50 ppb arsenic and this trend was also noted for *mt2* but was not statistically significant (Fig 5B and 5C). *ogg1* mRNA levels were also significantly lower at 48 hpf in the zinc deficient embryos treated with 500 ppb as compared to zinc adequate embryos exposed to 500 ppb arsenic (Fig 5D). At 120 hpf there was a significant difference in the *nrf2a* mRNA levels in zinc adequate and zinc deficient embryos at 50 ppb arsenic (Fig 5E). Additionally, *nrf2b* was consistently and significantly suppressed in zinc deficient embryos but there was no effect of arsenic exposure at this time point in development (Fig 5F). By 120 hpf neither zinc deficiency or arsenic exposure significantly impacted *mt2* and *ogg1* mRNA levels (Fig 5G and 5H). Similarly, *gclc* and *hmox1* mRNA levels were unchanged under any experimental conditions tested (data not shown).

**Fig 6:**
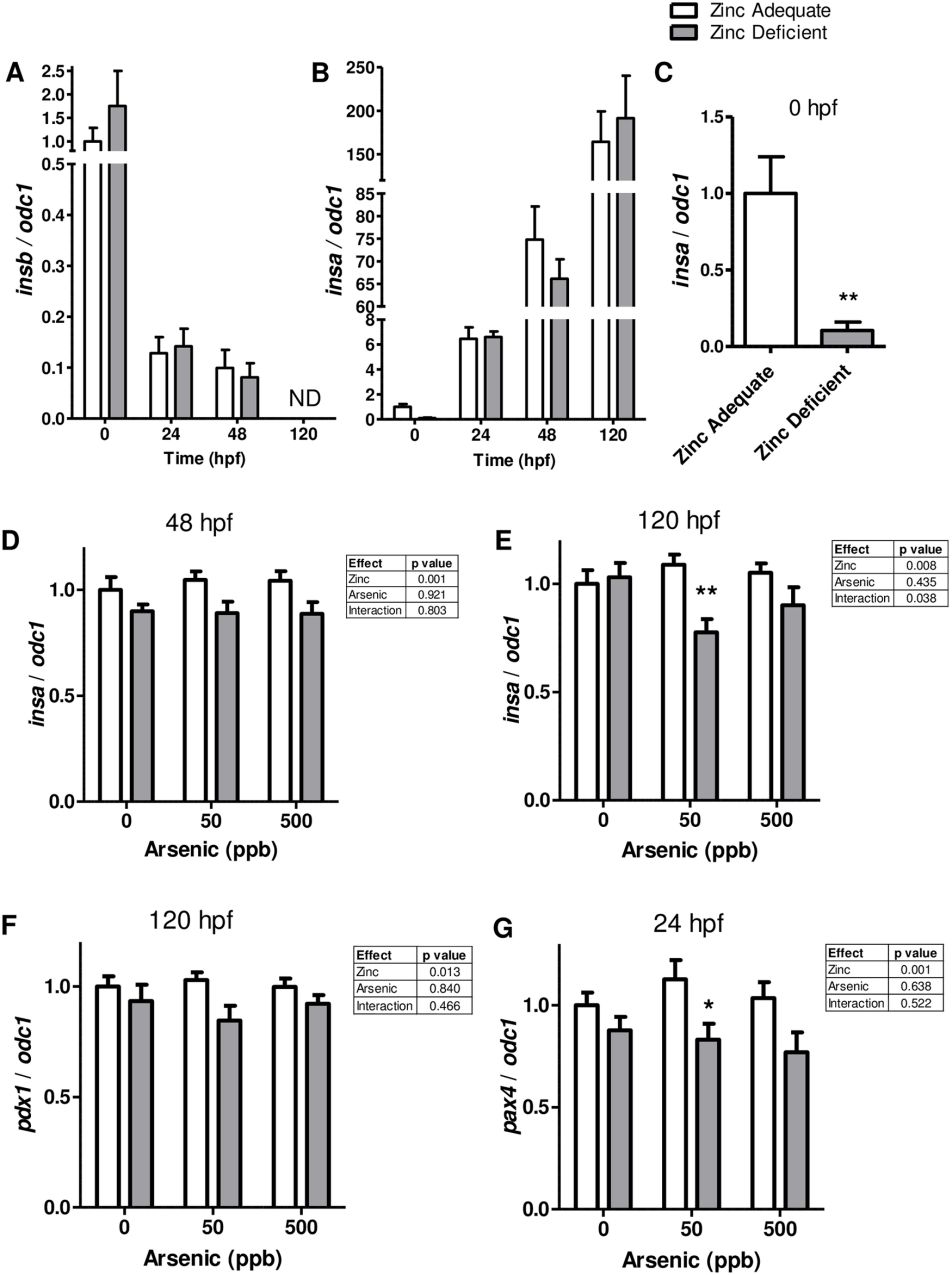
Effect of zinc deficiency and arsenic exposure on the mRNA levels of genes related to insulin production. A-G) Bars represent mean (± SEM) mRNA levels of the indicated genes in zinc adequate (white bars), or zinc deficient (grey bars). RNA was collected at the indicated time point and are from 3 independent experiments where n = 7–9. A-B) Bars show the dynamic nature of insulin gene expression over developmental time and ND denotes that transcript level was below detection limit. C) Significant difference between zinc adequate and zinc deficient *insa* mRNA levels at 0 hpf were calculated by t-test where ** indicates p < 0.01. (D-G) Significant differences between samples exposed to 0, 50, or 500 ppb arsenic were calculated using two-way ANOVAs with results detailed in individual tables for each gene and time point. Bonferroni post-tests were used to determine differences between zinc adequate and zinc deficient embryos at a given arsenic dose where * and ** indicate significant differences between the groups where p < 0.05 and p < 0.01 respectively.

We also examined the expression of the insulin gene because marginal maternal zinc status is associated with impaired glucose tolerance and increased susceptibility to diabetes [11]. It was also of interest because there is an increased risk for diabetes with long-term exposure to arsenic in the drinking water. Zebrafish have two genes that perform insulin function (*insa* and *insb*) which are dynamically expressed through out development from 0–120 hpf. *insb* is initially highly expressed and decreases over time (Fig 6A), while *insa* increases in expression over time (Fig 6B) [45]. Just after fertilization, *insa* mRNA levels were significantly suppressed (90%) in zinc deficient embryos (Fig 6C). At 24 hpf there was no significant effect of diet or arsenic exposure on *insa* mRNA levels. The difference between the findings at 0 hpf, and 24 hpf is likely related to the dynamic nature by which *insa* is turned on during development. At 48 hpf *insa* mRNA levels were modestly, but significantly decreased in zinc deficient embryos (Fig 6D). At 120 hpf the 50 ppb arsenic exposure caused the zinc deficient embryos to have a significant 29% decline in *insa* mRNA, relative to zinc adequate embryos also exposed to 50 ppb arsenic (Fig 6E). The two-way ANOVA shows both a significant effect of zinc deficiency on *insa* mRNA level, and a significant interaction between zinc deficiency and arsenic exposure at 120 hpf (Fig 6E). No significant difference in the *insb* mRNA levels was detected with zinc deficiency and/or arsenic exposure at 0–48 hpf, and as expected *insb* mRNA expression was not detectable at 120 hpf by qPCR (Fig 6A and data not shown).

Since insulin is made in pancreatic beta cells we next examined the expression of *paired box 4* (*pax4*) gene because it is a transcriptional regulator that is important in pancreatic islet beta cell differentiation and development [46]. We also examined the expression of the *pancreatic and duodenal homeobox 1* (*pdx1*) gene which regulates endocrine function and beta cell formation during pancreatic development [47, 48]. Pdx1 is also a key player in glucose-dependent regulation of insulin gene expression. *pdx1* mRNA levels were modestly but significantly suppressed in zinc deficient embryos at 120 hpf, but there was not a significant difference between the zinc adequate and zinc deficient embryos at specific arsenic concentrations (Fig 6D). We also found that *pax4* mRNA was significantly suppressed in zinc deficient embryos at 24 hpf (Fig 6E). Furthermore, at the 50 ppb arsenic exposure the zinc deficient embryos had a significant 26% reduction in *pax4* mRNA (Fig 6E).

## Discussion

It is estimated that one-third of the world’s population is at risk for zinc deficiency and this is a significant public health concern [49]. We do not have a complete understanding of the extent to which zinc deficiency may sensitize individuals to damage caused by environmental toxicants, particularly in a developing embryo where maternal zinc deficiency has been associated with adverse effects for the offspring. Here we used a zebrafish model and show for the first time that environmentally relevant concentrations of arsenic decreased the amount of zinc present in embryos. This is also the first study to show that the combination of a moderate zinc deficiency and arsenic exposure caused a significant decline in activity in the embryos, and this was greater than what was observed with either condition alone. Modest, but significant effects of zinc deficiency and/or arsenic exposure were also found in developing embryos on the expression of genes that regulate zinc transport, response to oxidative stress, and insulin production (including *zip1*, *znt7*, *nrf2*, *ogg1*, *pax4*, and *insa*). Zinc deficiency and arsenic exposures, often co-exist in the human population. Understanding how nutritional factors, like zinc, alter the susceptibility to environmental toxins will have a significant impact on risk assessment and strategies for remediation in susceptible communities.

Zinc is a tightly regulated element in the human body and the underlying mechanism by which zinc levels decrease in embryos exposed to arsenic is not known. It is possible that arsenic exposure increases the amount of zinc that is removed from the developing embryo via excretion (which is active at 48 hpf) and/or production of feces as the intestinal tract develops [50, 51]. At a cellular level arsenic exposure did not cause significant changes in expression of genes like *znt1*, which facilitate zinc efflux from cells [52]. A limitation to this work, and the results examining oxidative stress and insulin production genes, is that we evaluated only mRNA levels, and were unable to evaluate protein abundance. This is primarily because zebrafish embryos are very small and limited amount of tissue was available. Nevertheless, we did find a significant increase in *zip1* and *znt7* mRNA expression levels in zinc deficient embryos, as compared to zinc adequate controls. The difference in expression in zinc adequate and deficient embryos was lost with arsenic exposure because expression of *zip1* and *znt7* increased with arsenic in the zinc adequate embryos. Interestingly, zinc deficient embryos did not further upregulate *zip1* and *znt7* in the presence of arsenic and thus there was not a synergistic effect of zinc deficiency and arsenic exposure on these genes. It is of interest to examine why *zip1* and *znt7* expression is altered in the presence of arsenic to gain possible insights into the mechanism of how arsenic exposure alters zinc transport. Zip1 functions to increase cytoplasmic zinc concentrations and thus an increase in *zip1* mRNA in zinc adequate embryos is consistent with an increased need for zinc in the cells exposed to arsenic [53, 54]. Also, Znt7 protein transports zinc from the cytoplasm into the Golgi apparatus and facilitates the activation of zinc requiring enzymes [55]. As such, an increase in *znt7* with arsenic exposure could be indicative of a greater need for zinc in the Golgi apparatus. Previous work by others have shown that at the molecular level arsenic is known to displace zinc in proteins that contain specific types of zinc finger domains, like PARP-1 and XPA, which are important in DNA repair processes [56–58]. Because of this interaction arsenic inhibits the function of the proteins and promotes DNA damage particularly in the presence of a secondary carcinogen, like ultraviolet radiation [59, 60]. Due to the kinetics by which these two elements compete for binding to zinc finger pockets, zinc supplementation decreased the arsenic-enhanced DNA damage and suggests that zinc supplementation may be a useful strategy to improve DNA repair capacity in arsenic exposed populations [56, 57, 59, 60].

Epidemiology studies identified an increased risk of still births, miscarriages, birth defects, and infant mortality in people exposed to arsenic exposure [22–26]. Kile *et al*. also showed arsenic exposure during pregnancy was associated with lower birth weight, primarily because of a decrease in gestational age at birth [17]. We did not observe increased developmental defects or mortality in the embryo, at environmentally relevant 50 and 500 ppb arsenic concentrations. This is in agreement with previous zebrafish findings that showed the lowest observable effect level reported for arsenite was 500 μM (37.4 ppm) [61]. The differences between the fish and human literature may be related to differences in arsenic metabolism between the species [62]. Differences could also be related to the nature of external development of zebrafish eggs that may make them less susceptible to arsenic related problems in the mother that could lead to premature birth [17]. We did observe increased mortality in zinc deficient embryos and this is consistent with our previous report in zebrafish [34]. The combination of zinc deficiency and arsenic exposure did not cause a further increase in mortality or developmental malformations but did produce a significant ~40% reduction in activity, which was significantly greater than the hypoactivity observed with each condition individually. Decreased activity in this assay could be related to changes in various factors, including eye development, nerve and muscle function, and energy metabolism. An important area of future research would be to determine if the decreased activities observed in zinc deficient embryos is reversible, but it is beyond the scope of this current study. Future studies that restore zinc levels in the embryos, during specific windows of development, would be useful to determine if the decline in activity with zinc deficiency could be reversed. There is a body of literature linking zinc deficiency to altered neural function which could be contributing to the decreased movement observed with embryonic zinc deficiency. For example, reduced neurogenesis, and increased apoptosis have been found in the hippocampus of zinc deficient mice and zinc is known to modulate synaptic activity and neuronal plasticity [63, 64]. The decrease in activity in zinc deficient embryos is also consistent with our previous results in this model [34]. Neurotoxicity is well documented with arsenic exposure and could also be contributing to hypoactivity in exposed embryos [65]. In zebrafish embryos, ~1mM (75 ppm) arsenic has been associated with hypoactivity at 24 hpf and altered neural function with arsenic exposure has been observed [61]. A significant decline in motor function, cognition, and intellectual function has been found in children in Bangladesh who are exposed to arsenic at concentrations similar to those used here in their drinking water [66–69]. It has also been shown that zinc can protect neuronal cells grown in culture from arsenic induced cell death [70]. While we are unable to test cognition in zebrafish embryos, the hypoactivity we observe is consistent with the observation that arsenic exposure has adverse neurodevelopmental effects, and this could potentially be exacerbated by zinc deficiency. It is also possible that the arsenic-induced decline in zinc may also contribute to the decline in activity observed with arsenic exposure. Additional research examining the mechanisms leading to these phenotypes is an important future research priority. Overall the combination effect of zinc deficiency and arsenic on decreased activity of the embryo is worthy of further investigation ranging from metabolomics approaches in developmental models to epidemiology studies of affected populations. Some specific future directions should include, experiments limiting the window that embryos are exposed to arsenic. This could determine the specific periods during development that are most critical to alleviate arsenic exposure. Zinc supplementation experiments could evaluate if the decline in activity in zinc deficient embryos could be recovered later in life. Furthermore, zinc supplementation experiments would also be interesting in the context of arsenic exposed embryos to determine if increasing zinc levels could restore the arsenic induced deficit in activity.

Zinc deficiency and arsenic exposure are both conditions that are well documented to cause oxidative stress (reviewed in [21] and [42, 43]. Here we show in the zebrafish embryo that zinc deficiency was associated with the suppression of several genes that respond to oxidative stress (*nrf2a*, *nrf2b*, *mt2*, and *ogg1*) at the mRNA level. Suppression of *nrf2* with zinc deficiency has been observed in the past and has been associated with oxidative stress [71–73]. In contrast, Ogg1 protein has been shown to be induced with zinc deficiency in adult rat livers [42]. The difference between this published report and our observation of *ogg1* suppression may come from the level of expression evaluated (mRNA vs protein) and different species and tissues that were examined. Also, no difference in *mt2* expression was previously reported in the liver of mice pups born of mom’s fed control or low zinc diets [74]. However, zinc deficiency caused epigenetic modifications of *mt2* in the pups, causing overexpression of *mt2* mRNA when the pups were exposed to cadmium at 5 weeks of age [74]. The difference in our results regarding *mt2* expression could be due to the amount of zinc in the diet (3x less in the mouse paper), tissue examined (liver vs whole embryo), and age at which expression was evaluated. We also showed significant, but modest differences between zinc deficient and zinc adequate embryos exposed to some specific arsenic concentrations, but the effects generally did not persist into the later developmental time points. For this reason, we did not expand these studies to look at other endpoints related to oxidative stress, like lipid peroxidation, protein oxidation, or abundance of antioxidants or 8-oxoguanine. Nevertheless, an interesting report by Pineda *et al*. has shown that a combination of zinc, vitamin C, and vitamin E protected rat pups from oxidative stress (in the form of lipid peroxidation) induced by exposure to 50 ppm arsenic throughout pregnancy and lactation [75]. It would be of interest in future studies to evaluate if embryonic exposures to arsenic can cause similar changes in gene expression in adulthood. It is also worth noting that 50 ppb arsenic induced a significant decrease in nrf2a or nrf2b mRNA expression, while 500 ppb arsenic was not associated with this difference. A possible explanation for the dose dependent effect could be related to a differential effect of the embryos to the 10-fold difference in toxicant exposure. It is possible that zinc deficiency makes it harder to maintain nrf2 levels, particularly when a mild stress is present, like 50 ppb arsenic, but under the condition of higher levels of arsenic other compensatory pathways are activated in the zinc deficient samples making the expression similar to those seen in zinc adequate embryos also exposed to 500 ppb arsenic.

We also observed that zinc deficiency, and the combination of zinc deficiency and arsenic exposure, caused suppression of genes that regulate β cells development and insulin production in embryos. While the changes were usually modest, they may be physiologically relevant because the embryos were exposed to conditions that could happen in the human population (low doses of arsenic, and a 15% reduction in zinc levels). It is important to acknowledge that this finding is limited because we did not evaluate glucose or insulin levels in the embryos because of limited tissue availability, but the data is supported by literature showing that zinc is directly involved in insulin storage and secretion (reviewed in [12]). There is also a growing amount of literature linking zinc deficiency, and maternal zinc deficiency, to alterations in body composition, glucose tolerance, insulin response, and increased susceptibility to diabetic stress [11, 34, 76–79]. People exposed to low and moderate levels of arsenic are also at an increased risk for the development of type 2 diabetes, are more likely to have impaired glucose tolerance during pregnancy, and are more likely to develop gestational diabetes [80–83]. Together these results suggest that embryonic zinc deficiency, in combination with arsenic exposure, could influence the development of diabetes later in life. Given this important possibility, we are currently testing the extent to which zinc deficiency and arsenic exposure disrupts insulin production in cultured pancreatic β cells.

## Conclusions

Zinc deficiency and chronic low level arsenic exposures are both significant public health concerns that affect millions of people including pregnant women. Here we show for the first time that environmentally relevant concentrations of arsenic reduce the amount of zinc in a developing embryo. We also revealed a combined effect of embryonic zinc deficiency and arsenic exposure on activity of embryos, which was greater than what was found with either condition alone. We also found that zinc deficiency, or zinc deficiency and arsenic exposure together, caused significant changes in expression of genes that regulate zinc homeostasis, response to oxidative stress and insulin production. These results suggests that embryonic zinc deficiency and arsenic exposure have harmful effects on the developing embryo and may increase the risk for developing diseases like diabetes.

## Supporting information

S1 Fig Arsenic does not alter copper, iron, magnesium and selenium abundance in embryos. Data are the mean (± SEM) amount of the indicated element as determined by ICP-OES, in zinc adequate or zinc deficient embryos continuously exposed to 0, 50, or 500 ppb arsenic starting at 6 hpf and collected at 120 hpf. Data is representative of at least three independent experiments and n = 9–12. Data were analyzed for significant differences between samples using two-way ANOVAs and no significant results were found.
(PPTX)

S2 Fig Arsenic and zinc deficiency does not alter *zip3*, *zip4*, *zip6*, *zip7*, *zip9*, *zip10*, *zip11* and *zip13* mRNA levels in embryos. Bars represent mean (± SEM) mRNA levels of indicated zinc importers at 120 hpf in zinc adequate (white bars) or zinc deficient (grey bars) embryos exposed to 0, 50, or 500 ppb arsenic. Data represent an average of 7–8 replicates per treatment group and were obtained from 2 independent experiments. Data were analyzed for significant differences between samples using two-way ANOVAs and no significant results were found.
(PPTX)

S3 Fig Arsenic and zinc deficiency does not alter *znt1*, *znt2*, *znt4*, *znt5*, *znt6*, *znt8*, *znt9* and *mtf-1* mRNA levels in embryos. Bars represent mean (± SEM) mRNA levels of indicated zinc transporters and metal-regulatory transcription factor 1 (mtf-1) at 120 hpf in zinc adequate (white bars) or zinc deficient (grey bars) embryos exposed to 0, 50, or 500 ppb arsenic. Data represent an average of 7–8 replicates per treatment group and were obtained from 2 independent experiments. Data were analyzed for significant differences between samples using two-way ANOVAs and no significant results were found.
(PPTX)

S4 Fig Heatmap of developmental toxicity. Embryos were collected from zinc adequate or zinc deficient fish, exposed to 0, 50, or 500 ppb arsenic from 6–120 hpf and analyzed for the indicated developmental malformation or mortality. Data are plotted as percent incidence of each endpoint relative to total embryos evaluated at a given condition. Data are from five independent experiments and between 385 and 500 embryos were evaluated for each endpoint.
(TIF)

S1 Table qPCR primers. Primers utilized for quantification of mRNA levels are given.
(PPTX)

S2 Table Amount of zinc, calcium, copper, iron, magnesium and selenium in diet, adult fish and embryos. Data are mean values obtained by ICP-OES measurement in zinc adequate or zinc deficient diet, adult fish, and embryos. Diet samples are μg of the element / g of diet (n = 23). Adult samples are μg of the element / g of body weight (n = 35). Embryo samples are ng of the element / embryo, and were obtained 120 hpf (n = 34). Significant differences between the zinc adequate and zinc deficient samples were calculated using t-tests and *** indicate significant differences between the groups where p < 0.001 respectively.
(PPTX)

S3 Table Hypoactivity of zinc deficient and arsenic exposed embryos caused by decreased movement in the dark. Embryos were collected from zinc adequate or zinc deficient fish, exposed to 0, 50, or 500 ppb arsenic from 6–120 hpf and analyzed for larval activity. Locomotor activity of embryos at 120 hpf were measured by larval photomotor response assay and all data come from at least four independent experiments n = 228–329. The area under the curve for movement was calculated during either the light or dark phase of the assay and compared to the control embryos (zinc adequate with no arsenic exposure) using a Kolmogorov-Smirnov test. The exposure condition was considered significant if the P value was less than 0.01 and the percent change was greater than 10%.
(PPTX)
