# Supplementary figures and images for: Combinatorial effects of zinc deficiency and arsenic exposure on zebrafish (Danio rerio) development
Source: PLoS One. 2017 Aug 24;12(8):e0183831. doi: 10.1371/journal.pone.0183831 (PMC5570330; doi:10.1371/journal.pone.0183831)

## Slide 1
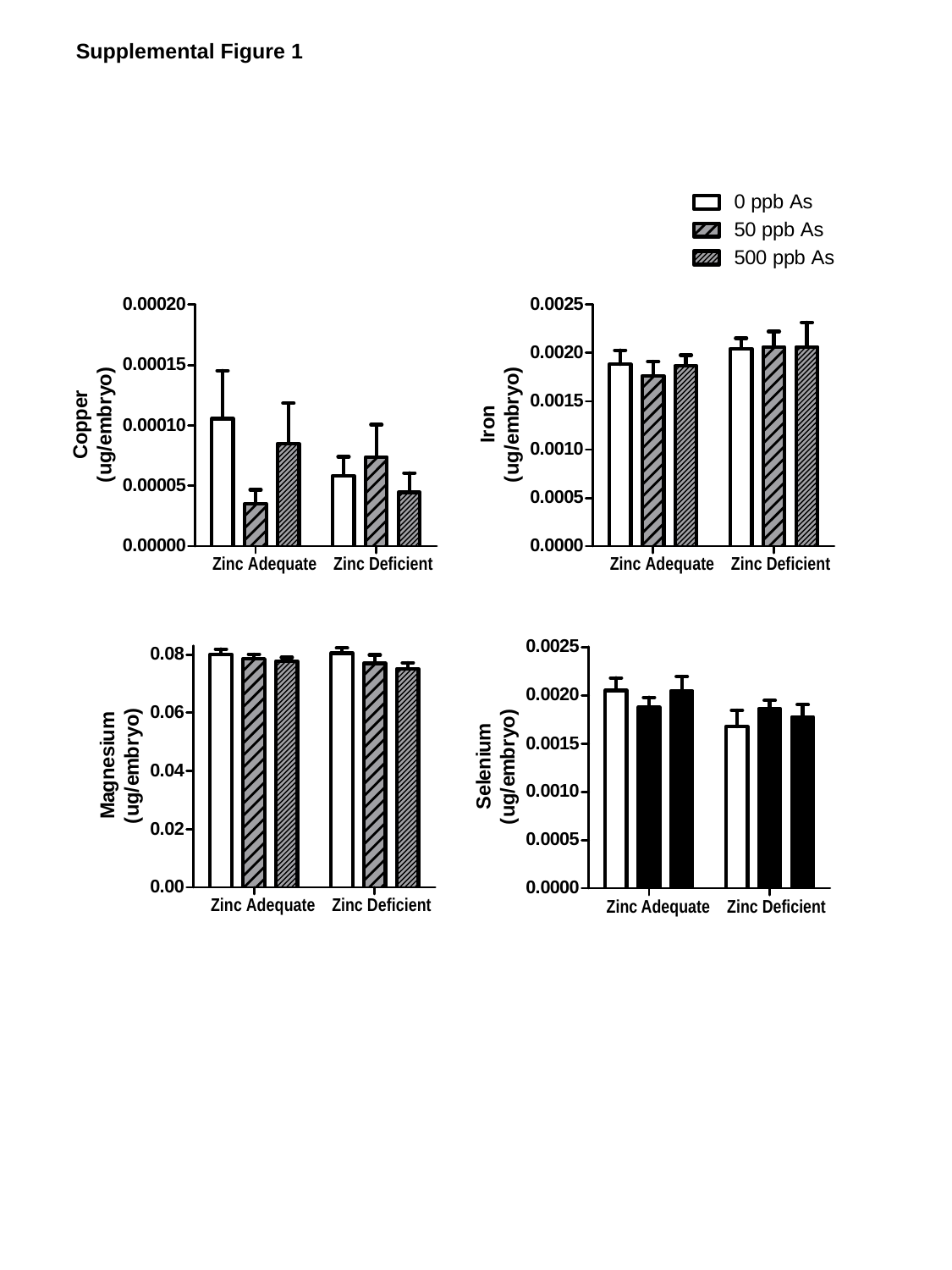

Supplemental Figure 1

Supplement: S1 Fig — Data are the mean (± SEM) amount of the indicated element as determined by ICP-OES, in zinc adequate or zinc deficient embryos continuously exposed to 0, 50, or 500 ppb arsenic starting at 6 hpf and collected at 120 hpf. Data is representative of at least three independent experiments and n = 9–12. Data were analyzed for significant differences between samples using two-way ANOVAs and no significant results were found. (PPTX) [file pone.0183831.s001.pptx]

## Slide 1
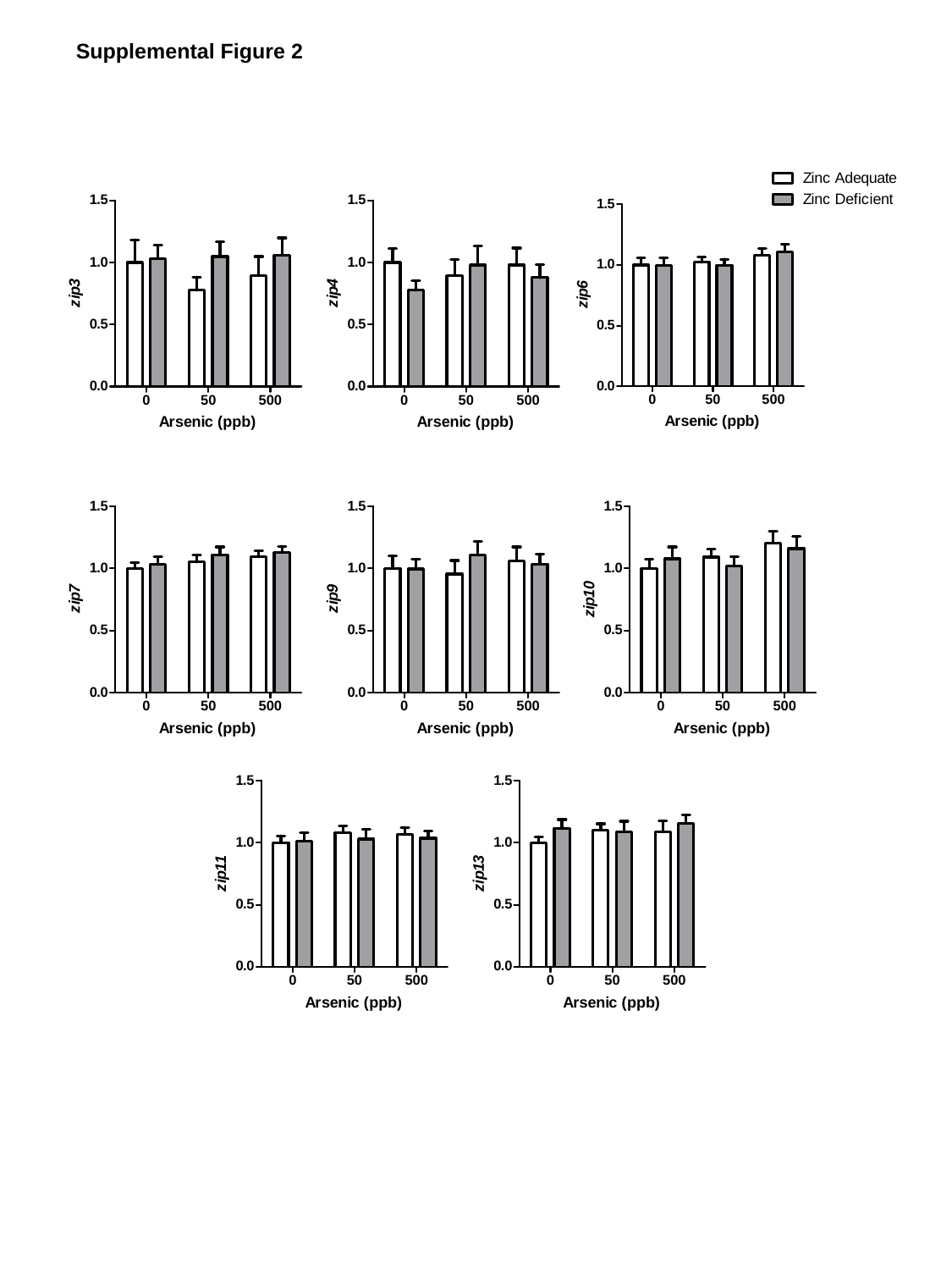

Supplemental Figure 2

Supplement: S2 Fig — Bars represent mean (± SEM) mRNA levels of indicated zinc importers at 120 hpf in zinc adequate (white bars) or zinc deficient (grey bars) embryos exposed to 0, 50, or 500 ppb arsenic. Data represent an average of 7–8 replicates per treatment group and were obtained from 2 independent experiments. Data were analyzed for significant differences between samples using two-way ANOVAs and no significant results were found. (PPTX) [file pone.0183831.s002.pptx]

## Slide 1
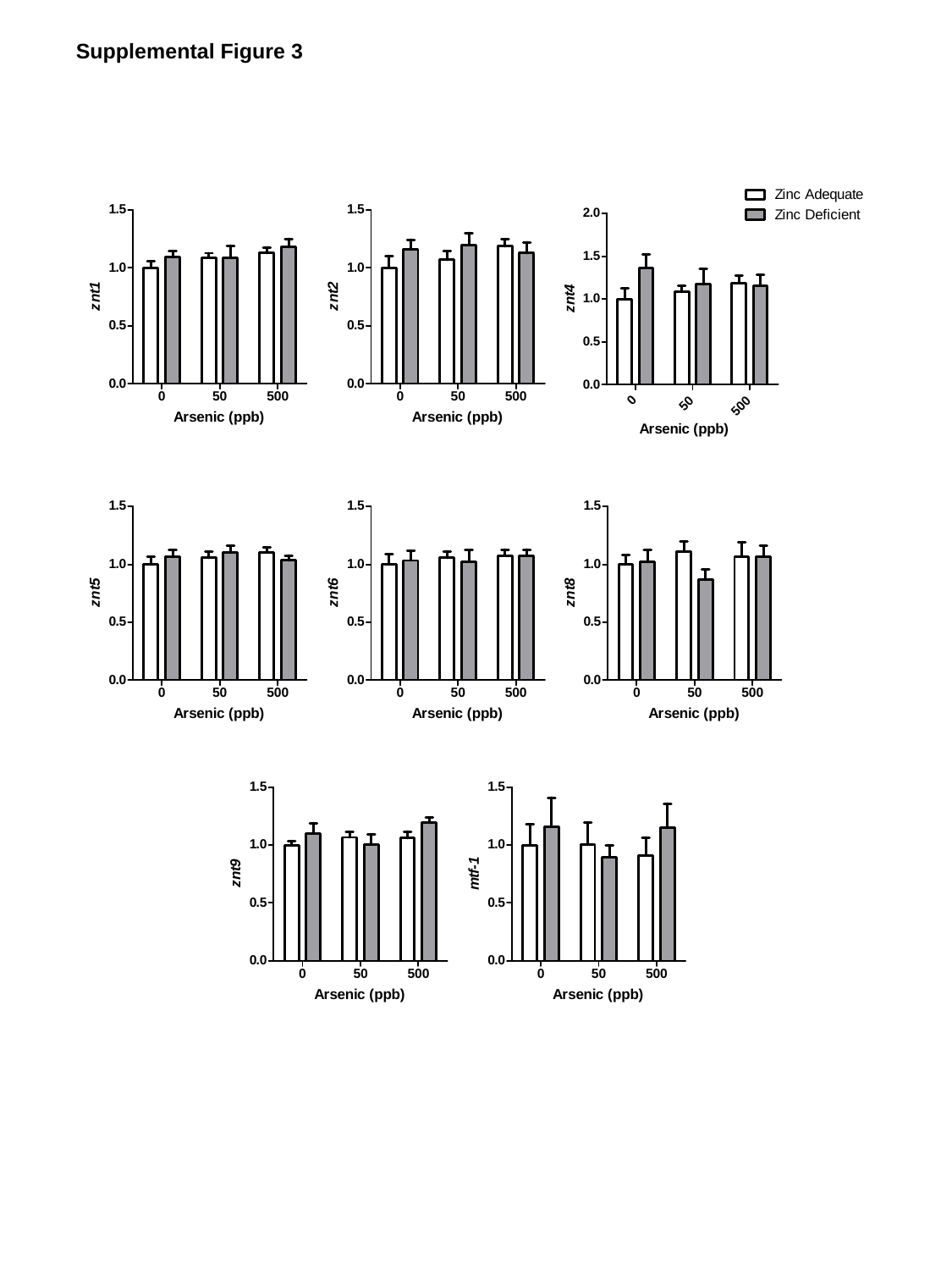

Supplemental Figure 3

Supplement: S3 Fig — Bars represent mean (± SEM) mRNA levels of indicated zinc transporters and metal-regulatory transcription factor 1 (mtf-1) at 120 hpf in zinc adequate (white bars) or zinc deficient (grey bars) embryos exposed to 0, 50, or 500 ppb arsenic. Data represent an average of 7–8 replicates per treatment group and were obtained from 2 independent experiments. Data were analyzed for significant differences between samples using two-way ANOVAs and no significant results were found. (PPTX) [file pone.0183831.s003.pptx]

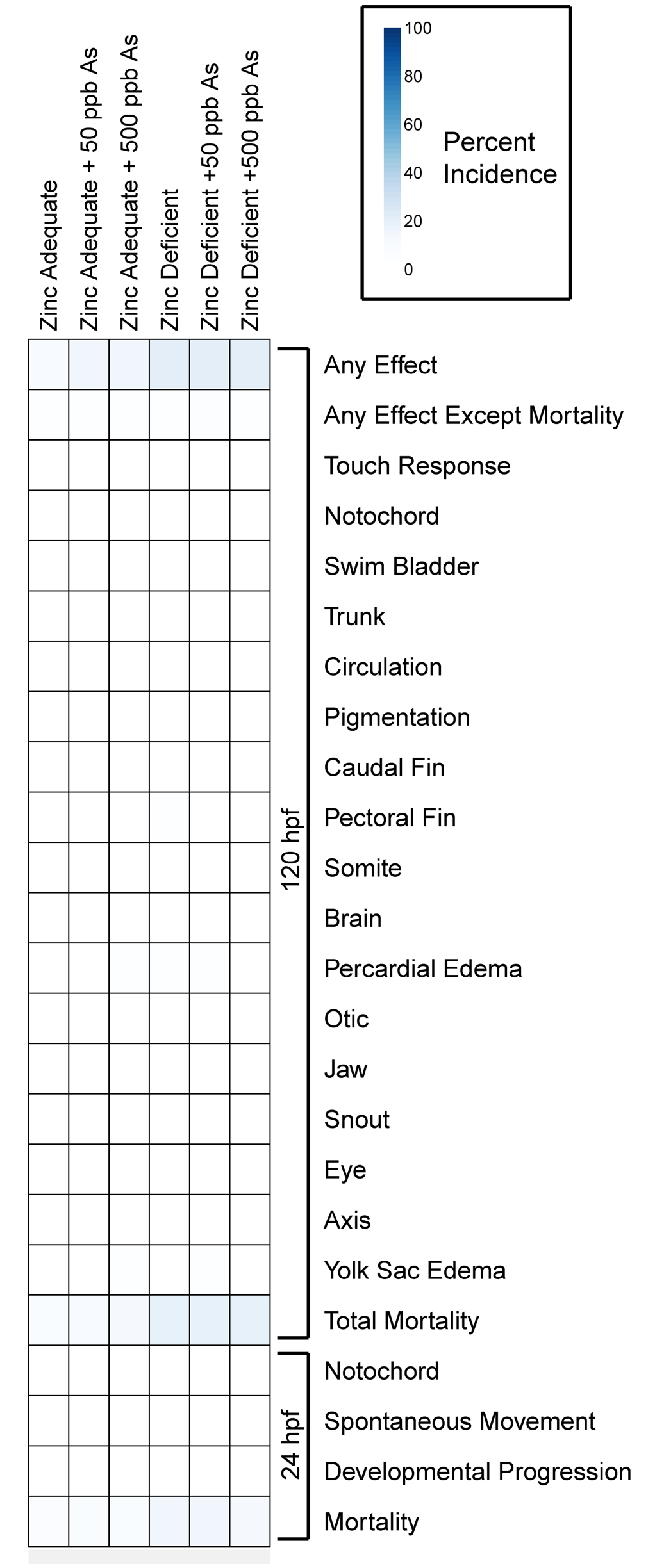

Supplement: S4 Fig — Embryos were collected from zinc adequate or zinc deficient fish, exposed to 0, 50, or 500 ppb arsenic from 6–120 hpf and analyzed for the indicated developmental malformation or mortality. Data are plotted as percent incidence of each endpoint relative to total embryos evaluated at a given condition. Data are from five independent experiments and between 385 and 500 embryos were evaluated for each endpoint. (TIF) [file pone.0183831.s004.TIF]

## Slide 1
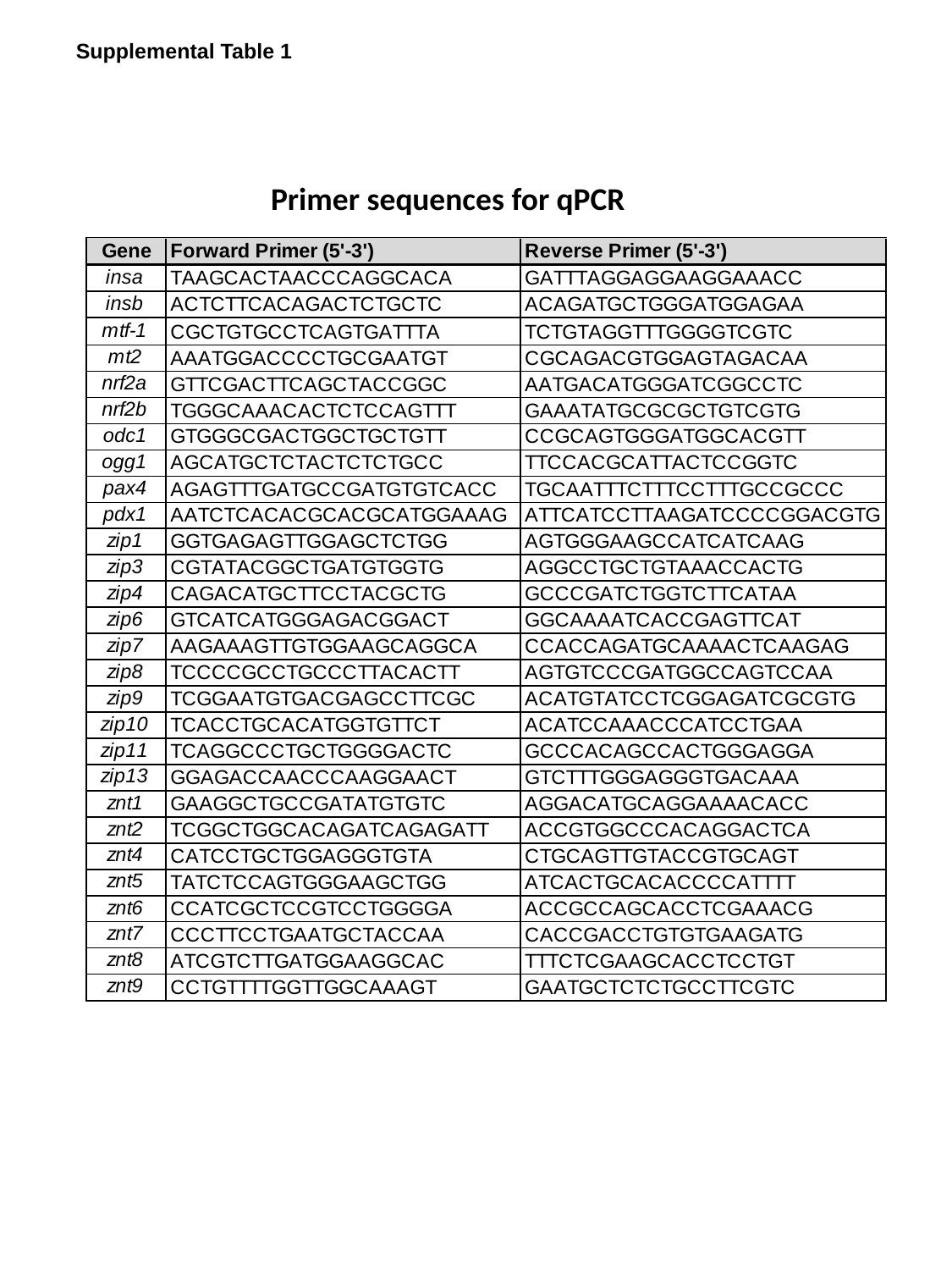

Supplemental Table 1
Primer sequences for qPCR

Supplement: S1 Table — Primers utilized for quantification of mRNA levels are given. (PPTX) [file pone.0183831.s005.pptx]
