# Supplementary material for: Combinatorial effects of zinc deficiency and arsenic exposure on zebrafish (Danio rerio) development
Source: PLoS One. 2017 Aug 24;12(8):e0183831. doi: 10.1371/journal.pone.0183831 (PMC5570330; doi:10.1371/journal.pone.0183831)
Supplement: S2 Table — Data are mean values obtained by ICP-OES measurement in zinc adequate or zinc deficient diet, adult fish, and embryos. Diet samples are μg of the element / g of diet (n = 23). Adult samples are μg of the element / g of body weight (n = 35). Embryo samples are ng of the element / embryo, and were obtained 120 hpf (n = 34). Significant differences between the zinc adequate and zinc deficient samples were calculated using t-tests and *** indicate significant differences between the groups where p < 0.001 respectively. (PPTX) [file pone.0183831.s006.pptx]

## Slide 1
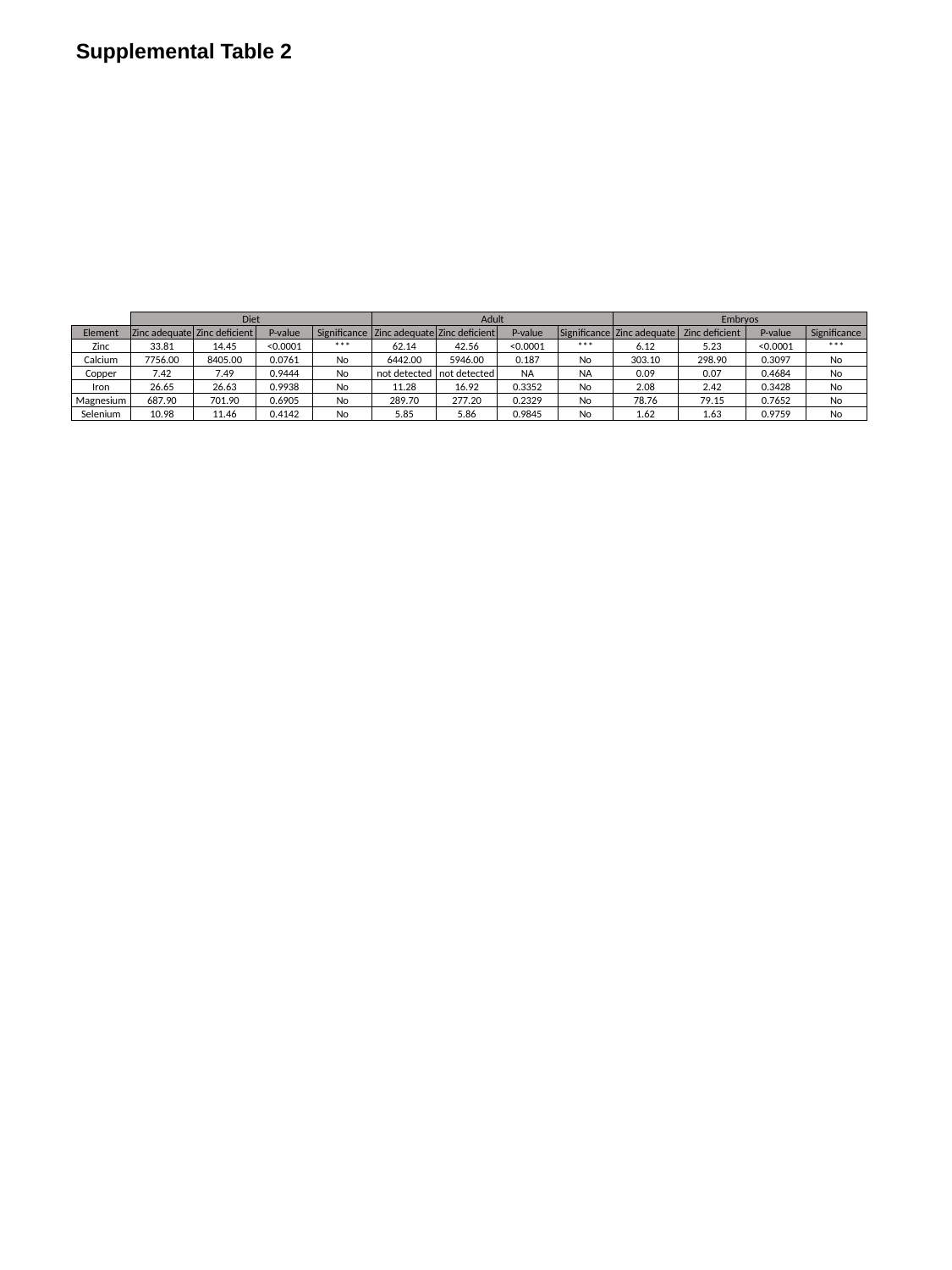

Supplemental Table 2
| | Diet | | | | Adult | | | | Embryos | | | |
| --- | --- | --- | --- | --- | --- | --- | --- | --- | --- | --- | --- | --- |
| Element | Zinc adequate | Zinc deficient | P-value | Significance | Zinc adequate | Zinc deficient | P-value | Significance | Zinc adequate | Zinc deficient | P-value | Significance |
| Zinc | 33.81 | 14.45 | <0.0001 | \*\*\* | 62.14 | 42.56 | <0.0001 | \*\*\* | 6.12 | 5.23 | <0.0001 | \*\*\* |
| Calcium | 7756.00 | 8405.00 | 0.0761 | No | 6442.00 | 5946.00 | 0.187 | No | 303.10 | 298.90 | 0.3097 | No |
| Copper | 7.42 | 7.49 | 0.9444 | No | not detected | not detected | NA | NA | 0.09 | 0.07 | 0.4684 | No |
| Iron | 26.65 | 26.63 | 0.9938 | No | 11.28 | 16.92 | 0.3352 | No | 2.08 | 2.42 | 0.3428 | No |
| Magnesium | 687.90 | 701.90 | 0.6905 | No | 289.70 | 277.20 | 0.2329 | No | 78.76 | 79.15 | 0.7652 | No |
| Selenium | 10.98 | 11.46 | 0.4142 | No | 5.85 | 5.86 | 0.9845 | No | 1.62 | 1.63 | 0.9759 | No |
