# Supplementary material for: Combinatorial effects of zinc deficiency and arsenic exposure on zebrafish (Danio rerio) development
Source: PLoS One. 2017 Aug 24;12(8):e0183831. doi: 10.1371/journal.pone.0183831 (PMC5570330; doi:10.1371/journal.pone.0183831)
Supplement: S3 Table — Embryos were collected from zinc adequate or zinc deficient fish, exposed to 0, 50, or 500 ppb arsenic from 6–120 hpf and analyzed for larval activity. Locomotor activity of embryos at 120 hpf were measured by larval photomotor response assay and all data come from at least four independent experiments n = 228–329. The area under the curve for movement was calculated during either the light or dark phase of the assay and compared to the control embryos (zinc adequate with no arsenic exposure) using a Kolmogorov-Smirnov test. The exposure condition was considered significant if the P value was less than 0.01 and the percent change was greater than 10%. (PPTX) [file pone.0183831.s007.pptx]

## Slide 1
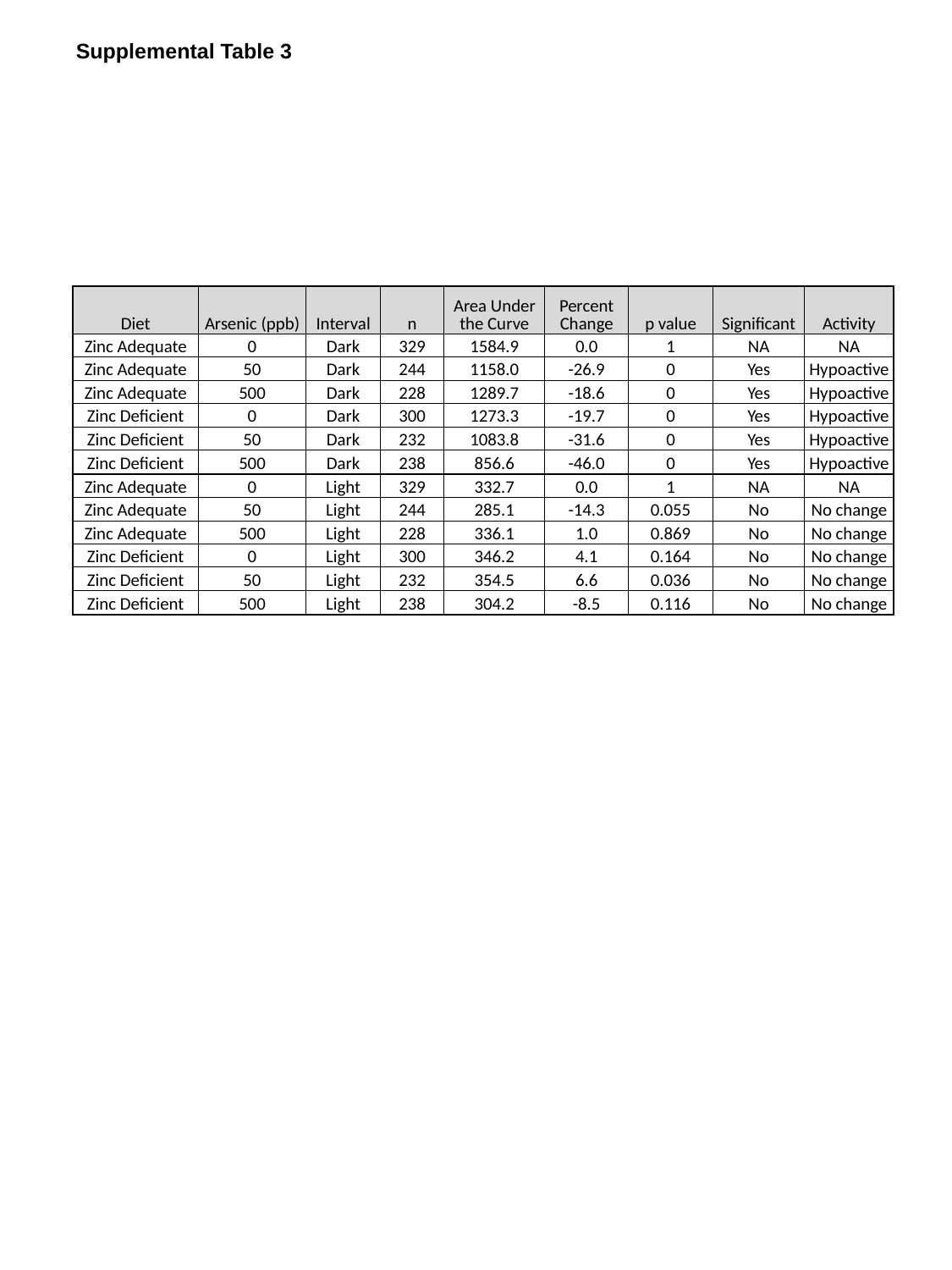

Supplemental Table 3
| Diet | Arsenic (ppb) | Interval | n | Area Under the Curve | Percent Change | p value | Significant | Activity |
| --- | --- | --- | --- | --- | --- | --- | --- | --- |
| Zinc Adequate | 0 | Dark | 329 | 1584.9 | 0.0 | 1 | NA | NA |
| Zinc Adequate | 50 | Dark | 244 | 1158.0 | -26.9 | 0 | Yes | Hypoactive |
| Zinc Adequate | 500 | Dark | 228 | 1289.7 | -18.6 | 0 | Yes | Hypoactive |
| Zinc Deficient | 0 | Dark | 300 | 1273.3 | -19.7 | 0 | Yes | Hypoactive |
| Zinc Deficient | 50 | Dark | 232 | 1083.8 | -31.6 | 0 | Yes | Hypoactive |
| Zinc Deficient | 500 | Dark | 238 | 856.6 | -46.0 | 0 | Yes | Hypoactive |
| Zinc Adequate | 0 | Light | 329 | 332.7 | 0.0 | 1 | NA | NA |
| Zinc Adequate | 50 | Light | 244 | 285.1 | -14.3 | 0.055 | No | No change |
| Zinc Adequate | 500 | Light | 228 | 336.1 | 1.0 | 0.869 | No | No change |
| Zinc Deficient | 0 | Light | 300 | 346.2 | 4.1 | 0.164 | No | No change |
| Zinc Deficient | 50 | Light | 232 | 354.5 | 6.6 | 0.036 | No | No change |
| Zinc Deficient | 500 | Light | 238 | 304.2 | -8.5 | 0.116 | No | No change |
